# A common reference population from four European Holstein populations increases reliability of genomic predictions

**DOI:** 10.1186/1297-9686-43-43

**Published:** 2011-12-12

**Authors:** Mogens S Lund, Adrianus PW de Roos, Alfred G de Vries, Tom Druet, Vincent Ducrocq, Sébastien Fritz, François Guillaume, Bernt Guldbrandtsen, Zenting Liu, Reinhard Reents, Chris Schrooten, Franz Seefried, Guosheng Su

**Affiliations:** 1 Aarhus University, Faculty of Science and Technology, Department of Molecular Biology and Genetics, AU-Foulum, PO Box 50, DK-8830 Tjele, Denmark; 2 CRV, P.O. Box 454, 6800 AL, Arnhem, the Netherlands; 3 INRA, UMR1313 Génétique Animale et Biologie Intégrative, F-78352 Jouy-en-Josas, France; 4 Institut de l'Elevage, 149 rue de Bercy, F-75595 Paris, France; 5 UNCEIA, 149 rue de Bercy, F-75595 Paris, France; 6 Unit of Animal Genomics, Faculty of Veterinary Medicine and Centre for Biomedical Integrative Genoproteomics, University of Liège, B-4000, Liège, Belgium; 7 VIT, Heideweg 1, 27283 Verden, Germany

## Abstract

**Background:**

Size of the reference population and reliability of phenotypes are crucial factors influencing the reliability of genomic predictions. It is therefore useful to combine closely related populations. Increased accuracies of genomic predictions depend on the number of individuals added to the reference population, the reliability of their phenotypes, and the relatedness of the populations that are combined.

**Methods:**

This paper assesses the increase in reliability achieved when combining four Holstein reference populations of 4000 bulls each, from European breeding organizations, i.e. UNCEIA (France), VikingGenetics (Denmark, Sweden, Finland), DHV-VIT (Germany) and CRV (The Netherlands, Flanders). Each partner validated its own bulls using their national reference data and the combined data, respectively.

**Results:**

Combining the data significantly increased the reliability of genomic predictions for bulls in all four populations. Reliabilities increased by 10%, compared to reliabilities obtained with national reference populations alone, when they were averaged over countries and the traits evaluated. For different traits and countries, the increase in reliability ranged from 2% to 19%.

**Conclusions:**

Genomic selection programs benefit greatly from combining data from several closely related populations into a single large reference population.

## Background

Genomic predictions rely on linkage disequilibrium between Single Nucleotide Polymorphisms (SNP) and polymorphisms in genes with effects on traits of interest. Linkage disequilibrium induces associations between SNP genotypes and phenotypes. SNP effects can then be estimated and combined to form genomic predictions. The accuracies of estimated SNP effects are expected to increase with the number and accuracy of available phenotypes. Therefore, the reliability of genomic predictions increases with the size of the reference population (RP) from which the relationship between phenotypes and SNP markers is determined [1,2]. Currently, a RP generally consists of genotyped and progeny tested bulls [1,2]. Because of the importance of the size of the RP, US and Canadian RP have been combined and it has been reported that exchanging data from reference populations is beneficial [3,4]. In European countries, the size of national Holstein RP is moderate, compared to that of the combined North American RP. In September 2009, four regional breeding organizations: UNCEIA (France), VikingGenetics (Denmark, Sweden, and Finland), DHV-VIT (Germany) and CRV (The Netherlands, Flanders) created a combined RP by contributing each 4000 bulls. The resulting enlarged joint European RP is expected to increase the reliabilities of genomic predictions considerably.

This study reports on the preliminary steps necessary to combine these four RP into a single one. It also assesses to what extent the combined RP improves genomic predictions by comparing the reliabilities of genomic predictions obtained with the combined and individual RP.

## Methods

### Joint genomic dataset

**Figure 1:**
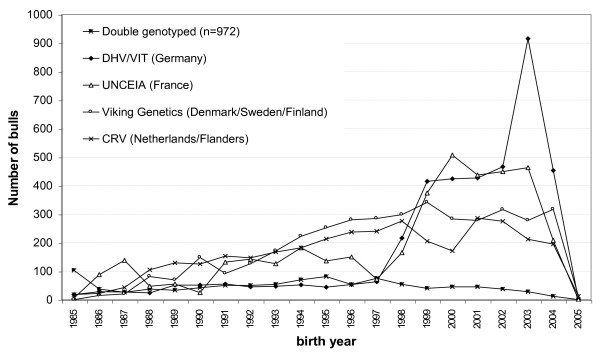
**Distribution of joint EuroGenomics reference bulls across birth years (n = 15966)**.

The joint dataset, hereafter called the EuroGenomics data, comprised 15966 progeny tested bulls. The distributions of the bulls in relation to birth year are plotted in Figure 1. Bulls provided by DHV-VIT and UNCEIA were predominantly born between 1999 and 2004, whereas those provided by VikingGenetics and CRV were predominantly born before 1999. Overall, the 15966 bulls had 19.4 million daughters, with 1389 bulls having more than 1000 daughters and 939 bulls having daughters in multiple countries. The average number of daughters per bull was 117, 85, 117 and 153 for bulls provided by DHV-VIT, UNCEIA, VikingGenetics and CRV, respectively.

### Imputation of genotypes across SNP chips

Genotypes provided by CRV were obtained using two versions of a custom 50 K SNP chip. They shared from 10 to 17 K SNP with the commercial Illumina BovineSNP50 chip [5] that was used to genotype the bulls of the three other partners. SNP genotypes unique to each chip were imputed by genotyping 972 influential bulls with both SNP chips, and applying a combination of programs, including DAGPHASE [6] and Beagle [7]. An independent cross-validation within the 972 genotyped bulls indicated that SNP genotypes were imputed with less than 1% error [8].

### Reference and validation data

Each partner validated its own bulls using the national RP and the EuroGenomics data. Deregressed proofs (DRP, [9,10]) calculated from EBV on the scale of the target population obtained from Interbull 2010-01 Multiple Across Country Evaluation (MACE) [11]) were used to predict and validate genomic predictions (GBV) of domestic bulls for three populations; for French Holsteins, daughter yield deviations (DYD) from the October 2009 national evaluation were used, because QTL mapping was already performed using these data. The national RP and EuroGenomics data were divided into reference and validation datasets by choosing a cut-off date for the birth date of bulls, so that approximately the 25% youngest national genotyped bulls were in the validation dataset. Records were included into the RP if the DRP/DYD had an effective daughter contribution (EDC) [12] of at least 20. A previous study [13] showed that reliabilities of genomic predictions for bulls whose sires were included in the reference population were much higher than for bulls without sires included. The proportion of bulls with their sires in the reference population differed among the four populations. Thus, to make results comparable, only the bulls whose sires were in the national RP were included in the validation data. In Germany, this criterion led to a significant decrease in the number of validation bulls. Thus, in order to increase the validation dataset for the German predictions, the German validation data included all bulls whose sire was included in the Eurogenomics RP when predictions were based on the EuroGenomics RP. The numbers of animals in the reference and validation datasets are in Table 1 for Denmark, Sweden and Finland (DFS), in Table 2 for Germany (DEU), in Table 3 for The Netherlands (NLD) and in Table 4 for France (FRA). Analyses were carried out for protein yield, udder depth, somatic cell score (SCS), and for female fertility as non-return rate (NRR) or interval from calving to first insemination (ICF).

**Table 1:** Validation of parent index (PI) and genomic breeding values (GBV) using Nordic (DSF_ref) and EuroGenomic (EU_ref) reference populations

Trait	Number of bulls			Nordic reference				EuroGenomic reference			
	DFS_ref	EU_ref	valid	REL_PI_	b_0_	b_1_	REL_GBV-PI_	REL_PI_	b_0_	b_1_	REL_GBV-PI_
Protein yield	3038	10701	942	0.21	2.63	0.82	0.19	0.22	1.98	0.86	0.32
Udder depth	2958	10755	948	0.12	2.80	0.98	0.29	0.13	1.70	0.90	0.42
SCS	3077	10880	947	0.21	1.27	0.99	0.19	0.22	0.44	0.94	0.32
NRR	3069	10712	942	0.29	-0.71	1.08	0.14	0.29	-1.02	0.98	0.19
Average	3035	10762	944	0.21	1.50	0.97	0.20	0.22	0.78	0.92	0.31

REL_PI_: r^2^_(PI, DRP)_/REL_DRP_, DRP is deregressed proofs in the validation dataset, REL_DRP _is the average reliability of DRP; REL_GBV-PI_: the difference between REL_GBV _and REL_PI_, where REL_GBV _is r^2^_(GBV, DRP)_/REL_(DRP)_; b_0 _is intercept and b_1 _is regression coefficient of DRP on GBV

**Table 2:** Validation of parent index (PI) and genomic breeding values (GBV) using German (DEU_ref) and EuroGenomic (EU_ref) reference populations

Trait	Number of bulls				German reference				EuroGenomic reference			
	**DEU ref**.	**DEU val**.	**EU ref**.	**EU val**.	REL_PI_	b_0 _/σ_g_	b_1_	REL_GBV-PI_	REL_PI_	b_0 _/σ_g_	b_1_	REL_GBV-PI_
Protein yield	3676	463	14475	1075	0.32	.29	0.83	0.28	0.32	.15	0.89	0.30
Udder depth	3672	454	14371	1048	0.22	-.08	0.97	0.26	0.20	-.16	1.01	0.45
SCS	3676	445	14479	1028	0.33	.04	0.83	0.26	0.33	.02	0.94	0.41
NRR	3676	314	14318	892	0.18	-.08	0.91	0.04	0.22	.11	0.91	0.14
Average	3675	419	14411	1011	0.26	.08	0.89	0.21	0.27	.03	0.94	0.32

REL_PI_: r^2^_(PI, DRP)_/REL_DRP_, DRP is deregressed proofs in the validation dataset, REL_DRP _is the average reliability of DRP; REL_GBV-PI _: the difference between REL_GBV _and REL_PI_, where REL_GBV _is r^2^_(GBV, DRP)_/REL_(DRP)_; b_0 _is intercept and b_1 _is regression coefficient of DRP on GBV; σ_g _is the genetic standard deviation

**Table 3:** Validation of parent index (PI) and genomic breeding values (GBV) using Dutch/Flemish (NDL_ref) and EuroGenomic (EU_ref) reference populations

Trait	Number of bulls			Dutch/Flemish reference				EuroGenomic reference			
	NLD_ref	EU_ref	valid	REL_PI_	b_0_/σ_g_	b_1_	REL_GBV-PI_	REL_PI_	b_0_/σ_g_	b_1_	REL_GBV-PI_
Protein yield	3471	9618	1115	0.25	0.02	0.99	0.23	0.24	0.01	0.94	0.28
Udder depth	3468	9541	1113	0.19	-0.04	1.00	0.19	0.19	-0.05	1.01	0.36
SCS	3458	9604	1107	0.29	-0.05	1.04	0.19	0.29	-0.06	1.06	0.27
ICF*	3472	9398	1117	0.35	0.09	1.03	0.18	0.33	0.10	1.03	0.21
Average	3467	9540	1113	0.27	0.01	1.02	0.20	0.26	0.00	1.01	0.28

*ICF: interval between calving and first insemination; REL_PI_: r^2^_(PI, DRP)_/REL_DRP_, DRP is deregressed proofs in the validation dataset, REL_DRP _is the average reliability of DRP; REL_GBV-PI_: the difference between REL_GBV _and REL_PI_, where REL_GBV _is r^2^_(GBV, DRP)_/REL_(DRP)_; b_0 _is intercept and b_1 _is regression coefficient of DRP on GBV; σ_g _is the genetic standard deviation

**Table 4:** Validation of parent index (PI) and genomic breeding values (GBV) using French (FRA_ref) and EuroGenomic (EU_ref) reference populations

Trait	French reference (3071 bulls)					EuroGenomic reference (12078 bulls)				
	REL_PI_	N_QTL_	b_0_/σ_g_	b_1_	REL_GBV-PI_	REL_PI_	N_QTL_	b_0_/σ_g_	b_1_	REL_GBV-PI_
Protein yield	0.23	206	0.25	0.79	0.17	0.24	324	0.19	0.79	0.21
Udder depth	0.16	216	0.05	0.96	0.23	0.14	310	-0.07	0.98	0.35
SCS	0.33	214	0.02	0.96	0.27	0.33	304	-0.02	0.95	0.35
CR*	0.24	166	0.11	0.79	0.14	0.22	280	0.09	0.85	0.24
Average	0.24	201	0.11	0.88	0.20	0.23	305	0.05	0.89	0.29

*CR: conception rate; REL_PI_: r^2^_(PI, DRP)_/REL_DRP_, DRP is deregressed proofs in the validation dataset, REL_DRP _is the average reliability of DRP; REL_GBV-PI_: the difference between REL_GBV _and REL_PI_, where REL_GBV _is r^2^_(GBV, DRP)_/REL_(DRP)_; b_0 _is intercept and b_1 _is regression coefficient of DRP on GBV; σ_g_: genetic standard deviation

### Genetic correlation between countries

The degree of genetic correlation for a given trait between countries reflects the importance of genotype by environment interactions. Table 5 shows for each population and each trait, the average genetic correlation with the three other populations, as obtained from INTERBULL [14]. These genetic correlations differed among countries and among traits. Among the traits studied here, udder depth had the highest genetic correlation between countries (0.98 on average), followed by protein yield (0.88) and SCS (0.88). Fertility had the lowest genetic correlation (0.70). The average genetic correlation of one country with the three other countries was highest for DFS and DEU (0.89), followed by FRA (0.85) and NLD (0.83).

**Table 5:** Average genetic correlation of a trait in a country with the same trait in the other three countries

	DEU	DFS	FRA	NLD	Average
Protein yield	0.870	0.883	0.877	0.883	0.878
Udder depth	0.977	0.983	0.977	0.983	0.980
SCS(U1)	0.890	0.897	0.903	0.823	0.878
Fertility	0.820	0.803	0.570	0.697	0.697
Average	0.889	0.892	0.832	0.847	0.858

### Statistical models

The four partners applied different genomic prediction models. The Nordic and German genomic predictions were obtained with a mixed linear model with random regression on coefficients of SNP genotypes, assuming equal variance of SNP effects over markers [15]. The Dutch/Flemish genomic predictions used a Bayesian mixture model for SNP effects, along with polygenic effects [16], assuming that most SNP had small effects and a few SNP had moderate or large effects and the French genomic predictions used a mixed linear model with a polygenic effect and random haplotype effects across the genome [17]. Included haplotypes were identified in an initial QTL detection step using LDLA [18] on the national RP. The QTL detection was carried out also with the EuroGenomics RP, but due to time constraints, the detection procedure used hidden states obtained from the Dualphase [6] software. Hence, two lists of QTL differing by the RP in which they were detected were used to estimate haplotype effects for the prediction models using the French or EuroGenomics RP, respectively. In all French analyses, 40% of the genetic variance was assumed to be explained by polygenes and 60% by markers. In all the models described above, the weighting factor, w = r^2^/(1-r^2^), was applied to account for heterogeneous residual variances due to different reliabilities of DRP (r^2^) or DYD.

### Validation criteria

Derivation of the GBV used for validation differed between partners. The Nordic validation was based on direct estimated genomic breeding values (DGV), as obtained from the genomic prediction model. The German validation combined DGV of the genotyped bulls and EBV of all available progeny-tested bulls to obtain a genomically enhanced breeding value (GEBV) using the approach reported by Ducrocq and Liu [19]. GBV in the Dutch/Flemish and French validations resembled GEBV, since their models included polygenic effects. The reliability of GBV (i.e. DGV or GEBV) was measured as the squared weighted correlation divided by the weighted mean of DRP (or DYD) reliabilities. The slope and intercept of weighted regressions of DRP on GBV for bulls in the validation dataset were also used to assess unbiasedness of the genomic predictions. The weights for these analyses were the same as those used for genomic prediction, but standardized such that the mean weight equals 1. In addition, reliability of the pedigree index (PI) for bulls in the test datasets was calculated using the data of bulls born before the cut-off date to divide reference and test datasets, but each partner based their calculations on different datasets. Germany and France calculated pedigree index (PI) based on national evaluation data (PI_1_) and on Interbull MACE proofs (PI_2_). The Nordic partner calculated PI_1 _from Nordic bulls and PI_2 _from all Interbull bulls but using Interbull MACE proofs, in both situations. In the Dutch/Flemish data, PI_1 _was calculated from the national reference data and PI_2 _from the EuroGenomics reference data, respectively. The gain in reliability attributed by the genomic information (REL_GBV-PI_) was calculated as the reliability of genomic breeding values (DGV or GEBV) minus the reliability of PI.

### Expected gains in reliability

Realized gains in reliability when the national RP was extended to the EuroGenomics RP were compared to the gains expected based on equations derived in Goddard and Hayes [20]. Factors such as the size of the national RP, the size of the EuroGenomics RP (which varies between populations), the average genetic correlations between traits measured within one country and in the other countries, and the reliability of DRP were taken into account.

## Results

### Reliability of DRP in the national and the EuroGenomics datasets

Reliabilities of DRP (or DYD in the case of France) in the reference dataset reflect the amount of phenotypic information available for each genotyped bull (Table 6). Although the heritability of SCS was much lower than that of protein yield and udder depth, the reliability of DRP for SCS was similar. Reliability of DRP for fertility was significantly lower than for the other traits, which is consistent with its very low heritability. Fertility is also the trait for which the reliability dropped most from the national RP to the EuroGenomics RP because the correlation between fertility traits among countries is lower than for the other traits. Reliabilities of DRP in the EuroGenomics reference data were generally lower than those in the national reference data. The difference in DRP reliabilities between the national and EuroGenomics data reflects the fact that genetic correlations between countries were less than one. Thus, the difference in DRP reliabilities between two datasets was largest for fertility.

**Table 6:** Heritability of the traits and average reliability of DRP in the national and the EuroGenomics reference datasets

Trait	DFS			DEU			NLD			FRA		
	h^2^	r^2^_DFS_	r^2^_EU_	h^2^	r^2^_DEU_	r^2^_EU_	h^2^	r^2^_NLD_	r^2^_EU_	h^2^	r^2^_FRA*_	r^2^_EU_
Protein yield	0.39	0.93	0.82	0.48	0.95	0.82	0.50	0.95	0.84	0.30	0.91	0.77
Udder depth	0.37	0.83	0.84	0.26	0.87	0.84	0.40	0.91	0.86	0.36	0.87	0.81
SCS	0.15	0.88	0.82	0.23	0.90	0.83	0.37	0.94	0.85	0.15	0.85	0.8
Fertility	0.02	0.58	0.48	0.02	0.65	0.56	0.22	0.90	0.72	0.02	0.61	0.39
Average	0.23	0.81	0.74	0.25	0.84	0.76	0.37	0.93	0.82	0.21	0.81	0.69

*DYD used instead of DRP

### Nordic validation

For the DFS reference population, substantial increases were observed in REL_G-PI_, when using the EuroGenomics data instead of the national data (Table 1). On average, the reliability of DGV was 20% higher than the reliability of PI in the DFS reference population. The average increase in REL_GBV-PI _obtained by going from the national to the EuroGenomics data was 11%. The largest benefits from using the EuroGenomics instead of the national data were observed for protein yield, udder depth and SCS. The coefficients of regression of DRP on DGV ranged from 0.82 to 1.08, and the intercepts were between -1.02 and 2.80 genetic standard deviation units.

### German validation

Averaged over all traits, the reliability of GEBV from the German RP was 21% higher than the reliability of PI_1 _(Table 2). The smallest increase was observed for NRR. The reliability of GEBV from the EuroGenomics data was 32% higher than the reliability of PI_2_. REL_GBV-PI _from the EuroGenomics data averaged over all traits was 11% higher than REL_GBV-PI _from the national reference dataset. The coefficients of regression of DRP on GEBV varied from 0.83 to 1.01, and the intercepts ranged from -0.16 to 0.29 genetic standard deviation units.

### The Dutch/Flemish validation

REL_GBV-PI _computed from the EuroGenomics data were on average 8% higher than those from the national data (Table 3). Reliabilities of GEBV were on average 20% higher than reliabilities of PI. In line with the Nordic validation, the largest benefits from using the EuroGenomics data were observed for protein yield, udder depth and SCS. The coefficients of regression of DRP on GEBV were around unity (0.94 - 1.06). In genetic standard deviation units, the intercepts ranged from -0.06 to 0.10.

### French validation

The reliability of GEBV was significantly higher than the reliability of PI for all traits (Table 4). Averaged over the four traits, the reliability of GEBV obtained from the EuroGenomics data was 9% higher than that from the national data. The latter was 20% higher than the reliability of PI. The coefficients of regression of DRP on GEBV were between 0.79 and 0.98; the intercepts were in the range of -0.07 to 0.25 genetic standard deviation units.

### Realized and expected gains in reliabilities from enlarged reference data

Realized and expected gains in reliabilities of genomic predictions when going from national to EuroGenomics data varied between traits and populations (Table 7). Expected gains increased over traits from fertility (lowest), protein yield, SCS to udder depth. Averaged over the four populations, the realized gains followed the same order, except for protein yield, which ranked second for expected gain but realized the lowest gain. This low outcome was observed in all the populations, except for DFS. For udder depth, high gains were generally achieved, especially for DEU and NLD. For SCS, the increase was generally high and was larger for DFS and DEU than for NLD and FRA. For fertility, DEU and FRA achieved larger gains than DFS and NLD.

**Table 7:** Realized and (expected) increases in reliability of genomic predictions when going from national to using EuroGenomics reference population

	DFS	DEU	NLD	FRA	Average
Protein yield	0.13 (0.12)	0.02 (0.13)	0.05 (0.07)	0.04 (0.13)	0.06 (0.11)
Udder depth	0.13 (0.16)	0.19 (0.15)	0.17 (0.08)	0.12 (0.15)	0.15 (0.14)
SCS	0.13 (0.13)	0.15 (0.14)	0.08 (0.08)	0.08 (0.16)	0.11 (0.13)
Fertility	0.05 (0.11)	0.10 (0.14)	0.03 (0.05)	0.10 (0.08)	0.07 (0.10)
Average	0.11 (0.13)	0.12 (0.14)	0.08 (0.07)	0.09 (0.13)	0.10 (0.12)

Averaged over traits, the expected gains by population increased in the following order: NLD, DFS, FRA, and DEU. The order of the realized gains was the same, except for FRA, which had the second largest expected gain, but only ranked third highest in realized gain.

## Discussion

Combining reference datasets, the reliability of genomic predictions, averaged over four populations and four traits, increased by 10% compared to genomic predictions using national RP alone. This demonstrates the benefit of combining four European Holstein RP into a single EuroGenomics RP. The size of the RP is one of the most important factors affecting the accuracy of genomic predictions. Currently, the RP generally consists of bulls which have already gone through a progeny test program. Goddard and Hayes [21] demonstrated that even for a trait for which the response variable has a reliability of 0.80 (such as DRP of progeny tested bulls for most traits) and a RP with 20000 individuals, reliabilities can be increased by further increasing the size of the RP. At present, no single country has a RP large enough to obtain the maximum accuracy of genomic prediction.

The magnitude of the expected increases in reliabilities from combining RP varied between the four partners and the four traits. The factors that explain most of this variation are differences in the actual increase in RP size and differences in reliabilities of DRP/DYD based on national and EuroGenomics data. The differences in reliabilities of foreign DRP are a consequence of differences in genetic correlations between countries (reflecting genotype by environment interactions), and differences in heritability and the number of daughters in the DRP. In general, the observed increases in reliabilities from combining RP were in line with the expected values (Table 7).

### Different gains among countries

The average increase in reliability of genomic prediction was 11% for DEU, 11% for DFS, 9% for FRA and 8% for NLD. This trend was consistent with expectations, except for France, which had the highest expected gain but only the third highest realized gain. The main factor generating the differences in the expected increase in reliability was the increase in the number of bulls in the reference populations. The cut-off points for dividing the EuroGenomics data into the reference dataset and the validation dataset differed between the four partners in order to meet the requirement that the size of the validation data should be about 25% of that of the national dataset. This was due to large differences in the age distribution of bulls in the different populations. Consequently, the differences between the size of national and EuroGenomics RP varied considerably (Tables 1, 2, 3 and 3). This led to increases in the size of RP reaching 10736, 7727, 9007 and 6073 for DEU, DFS, FRA and NLD, respectively. The expected gain was similar between DFS and FRA even though the RP increased more for FRA. One explanation is that FRA had the lowest average trait genetic correlations with the other three countries. The average genetic correlation between France and the other partners was only 0.57 for fertility. This is a consequence of FRA using CR rather than the NRR that is used by the other partners. These correlations are directly related to the accuracy of DRP of foreign bulls on the national scale, which is causing different gains in reliabilities among the countries. The increase in reliability deviated most from expectations for France, where the gain was less than expected. France uses the most complicated procedure to predict GBV, including a QTL detection step and inclusion of haplotypes for which a likelihood ratio test exceeds a predefined liberal threshold. This detection step was only performed on the national RP, so the EuroGenomics RP was not exploited to select which marker haplotypes were used in the final model. This is probably the main reason why France does not appear to reach the full potential of using the EuroGenomics versus the national RP.

### Different gains among traits

Among the four traits in this study, using the EuroGenomics data improved reliabilities of genomic predictions most for udder depth, followed by SCS, protein yield and fertility. This order of improved genomic predictions is consistent with expectations, with the exception of protein yield. The reason why the largest gain was observed for udder depth (12-19%) is largely due to the very strong genetic correlation between countries (0.98) for this trait. Average genetic correlations between countries were 0.88 for both SCS and protein yield but the average gain in reliability from using the EuroGenomics data was 11% for SCS but only 6% for protein yield This might be explained by the fact that the reliability of DRP in the EuroGenomics data was much lower than that in the national data for protein yield, while differences in reliabilities were smaller for SCS. In other words, the EuroGenomics data provide more information for SCS but less information for protein yield.

Generally, traits with a low heritability are expected to benefit relatively more from a larger reference population. However, in this study a relatively low gain was observed for fertility. The most likely reasons are that fertility had a low genetic correlation (in part due to differences in trait definitions) between countries and that reliability of DRP was much lower in the EuroGenomics data than in the national data. This is reflected in the calculated expectations of increased reliabilities, which is why fertility was also expected to show the lowest increase.

Longevity was not included in the analyses although it is an important trait in all breeding goals, because the definition of longevity differs substantially between countries. Our aim was to study the increase of reliabilities from combining training data for traits with different heritabilities (low for fertility, medium for SCS, and high for udder depth and protein yield) and different ranges of genetic correlations between countries (low for fertility, medium for SCS and protein yield, and highest for udder depth).

### Genomic prediction using national reference populations

In the present study, the sizes of the four national reference datasets were almost the same and the reliabilities of DRP were also similar, but prediction models used by the EuroGenomics partners were different. Previous simulation studies e.g. [22-24] showed that variable selection models (e.g., BayesB) have a greater predictive ability than models allowing for weaker differentiation of variances among markers (e.g., BayesA), and the latter were superior to linear BLUP models. However, based on real data from dairy cattle, VanRaden et al. [2] reported that the predictive ability of a nonlinear BLUP model (a heavy-tailed prior model) was considerably better than a linear BLUP model for fat percentage and protein percentage, while their predictive abilities were similar for 25 other traits. Cole et al. [25] reported that a heavy-tailed prior (analogous to BayesA) provided a slightly higher GEBV reliability for all nine traits than a finite locus model with heavy tails (analogous to BayesB) and higher than a linear model for fat yield, fat % and protein %. Su et al. [26] reported that a common prior Bayesian model (analogous to BayesA) exhibited a greater predictive ability than a mixture prior Bayesian model (analogous to BayesB) for fertility, udder health and protein yield, but not for fat %. In the present study, DEU and DFS used a linear BLUP model (random regression on SNP), NLD applied a Bayesian mixture model including polygenic effects, and FRA used a mixed linear model including pre-selected haplotypes and polygenic effects. Although applying different prediction models, the gains from genomic prediction over a conventional pedigree index using national reference data were similar between countries. Averaged over the four traits, the reliability of predicted breeding values was increased by 20-21% for the four partners. This suggests that the different models used in this study had a similar predictive ability.

### Measure of the reliability of genomic prediction

In this study, reliabilities of DGV, GEBV and PI were measured as the squared correlation divided by reliability of DRP for bulls in the validation data. This measure of reliability is unbiased only if the validation bulls come from a random sample but the bulls in this study were selected on the basis of PI. Directional selection is expected to reduce the correlation between PI (also DGV and GEBV) and DRP. Therefore, the reliabilities reported in this study might underestimate the reliability for a random group of bulls, especially for strongly selected traits. This underestimation could partly explain the difference in the presented PI reliability among the countries, as the selection intensities on the validation data could differ between countries. The amount of underestimation of reliability from the current validation might be similar to the difference (D_PI_) between the expected reliability of PI estimated by traditional BLUP based on the whole population and the reliability of PI estimated from the validation-based selected data. Thus, estimates of the reliability of DGV and GEBV for an unselected population are approximately equal to the reported reliability in the current validation plus D_PI _[2].

## Conclusions

This study showed that reliabilities of genomic predictions using EuroGenomics data were considerably higher than those using national reference data alone. The results confirm the importance of the size of reference populations for genomic prediction. A significant improvement of genomic prediction can be achieved through cooperation between countries by combining reference data.

## Competing interests

The authors declare that they have no competing interests.

## Authors' contributions

MSL, AR, TD, VD, BG, ZL CS, and GS carried out data exchange and analysis. MSL drafted the manuscript. All authors conceived of the study, and participated in its design. All authors read and approved the final manuscript.

## References

[B1] Hayes BJ Bowman PJ Chamberlain AJ Goddard ME Invited review: Genomic selection in dairy cattle: Progress and challenges J Dairy Sci 2009 92 433 443 10.3168/jds.2008-1646 19164653

[B2] VanRaden PM Van Tassell CP Wiggans GR Sonstegard TS Schnabel RD Taylor JF Schenkel FS Invited review: Reliability of genomic predictions for North American Holstein bulls J Dairy Sci 2009 92 16 24 10.3168/jds.2008-1514 19109259

[B3] Schenkel FS Sargolzaei M Kistemaker G Jansen GB Sullivan P Van Doormaal BJ Van Raden PM Wiggans GR Reliability of genomic evaluation of Holstein cattle in Canada Interbull Bull 2009 39 51 58

[B4] Cromie AR Berry DP Wickham B Kearney JF Pena J van Kaam JBCH Gengler N Szyda J Schnyder U Coffey M Moster B Hagiya K Weller JI Abernethy D Spelman R International genomic co-operation; Who, what, when, where, why and how? Interbull Bull 2010 42 72 78

[B5] Matukumalli LK Lawley CT Schnabel RD Taylor JF Allan MF Heaton MP O'Connell J Moore SS Smith TPL Sonstegard TS Van Tassell CP Development and characterization of a high density SNP genotyping assay for cattle PLoS ONE 2009 4 e5350 10.1371/journal.pone.0005350 19390634 PMC2669730

[B6] Druet T Georges M A hidden Markov model combining linkage and linkage disequilibrium information for haplotype reconstruction and quantitative trait locus fine mapping Genetics 2010 184 789 798 10.1534/genetics.109.108431 20008575 PMC2845346

[B7] Browning SR Browning BL Rapid and accurate haplotype phasing and missing-data inference for whole-genome association studies by use of localized haplotype clustering Am J Hum Genet 2007 81 1084 1097 10.1086/521987 17924348 PMC2265661

[B8] Druet T Schrooten C de Roos S In silico genotyping of thousands of SNP in dairy cattle for the eurogenomics project Proceedings of the 9th World Congress on Genetics Applied to livestock: 1-6 August 2010; Leipzig 2010 Gesellschaft für Tierzuchtwissenschaften e.V 137

[B9] Jairath L Dekkers JCM Schaeffer LR Liu Z Burnside EB Kolstad B Genetic evaluation for herd life in Canada J Dairy Sci 1998 81 550 562 10.3168/jds.S0022-0302(98)75607-3 9532510

[B10] Schaeffer LR Multiple trait international bull comparisons Livestock Prod Sci 2001 69 145 153 10.1016/S0301-6226(00)00255-4

[B11] Schaeffer LR Multiple-country comparison of dairy sires J Dairy Sci 1994 77 2671 2678 10.3168/jds.S0022-0302(94)77209-X 7814738

[B12] Fikse WF Banos G Weighting factors of sire daughter information in international genetic evaluations J Dairy Sci 2001 84 1759 1767 10.3168/jds.S0022-0302(01)74611-5 11467826

[B13] Lund MS Su G Nielsen US Aamand GE Relation between accuracies of genomic predictions and ancestral links to the training data Interbull Bull 2009 40 162 166

[B14] INTERBULL, International bull evaluation service http://www.interbull.org

[B15] VanRaden PM Efficient methods to compute genomic predictions J Dairy Sci 2008 91 4414 4423 10.3168/jds.2007-0980 18946147

[B16] Calus MPL Meuwissen THE de Roos APW Veerkamp RF Accuracy of genomic selection using different methods to define haplotypes Genetics 2008 178 553 561 10.1534/genetics.107.080838 18202394 PMC2206101

[B17] Ducrocq V Fritz S Guillaume F Boichard D French report on the use of genomic evaluation Interbull Bull 2009 39 17 21

[B18] Druet T Fritz S Boussaha M Ben-Jemaa S Guillaume F Derbala D Zelenika D Lechner D Charon C Boichard D Gut IG Eggen A Gautier M Fine mapping of quantitative trait loci affecting female fertility in dairy cattle on BTA03 using a dense single-nucleotide polymorphism map Genetics 2008 178 2227 2235 10.1534/genetics.107.085035 18430945 PMC2323811

[B19] Ducrocq V Liu Z Combining genomic and classical information in national BLUP evaluations Interbull Bull 2009 40 172 177

[B20] Goddard ME Genomic selection: prediction of accuracy and maximisation of long term response Genetica 2009 136 245 257 10.1007/s10709-008-9308-0 18704696

[B21] Goddard ME Hayes BJ Mapping genes for complex traits and their use in breeding programs Nat Rev Genet 2009 10 381 391 10.1038/nrg2575 19448663

[B22] Meuwissen THE Hayes BJ Goddard ME Prediction of total genetic value using genome wide dense marker maps Genetics 2001 157 1819 1829 11290733 10.1093/genetics/157.4.1819 PMC1461589

[B23] Lund MS Sahana G de Koning DJ Su G Carlborg Ö Comparison of analyses of the QTLMAS XII common dataset. I: Genomic selection BMC Proc 2009 3 S1 19278535 10.1186/1753-6561-3-s1-s1 PMC2654490

[B24] Guo G Lund MS Zhang Y Su G Comparison between genomic predictions using daughter yield deviation and conventional estimated breeding value as response variables J Anim Breed Genet 2010 127 423 432 10.1111/j.1439-0388.2010.00878.x 21077966

[B25] Cole JB VanRaden PM O'Connell JR Van Tassel CP Sonstegard TS Schnabel RD Taylor JF Wiggans GR Distribution and location of genetic effects for dairy traits J Dairy Sci 2009 92 2931 2946 10.3168/jds.2008-1762 19448026

[B26] Su G Guldbrandtsen B Gregersen VR Lund MS Preliminary investigation on reliability of genomic estimated breeding values in the Danish and Swedish Holstein Population J Dairy Sci 2010 93 1175 1183 10.3168/jds.2009-2192 20172238

